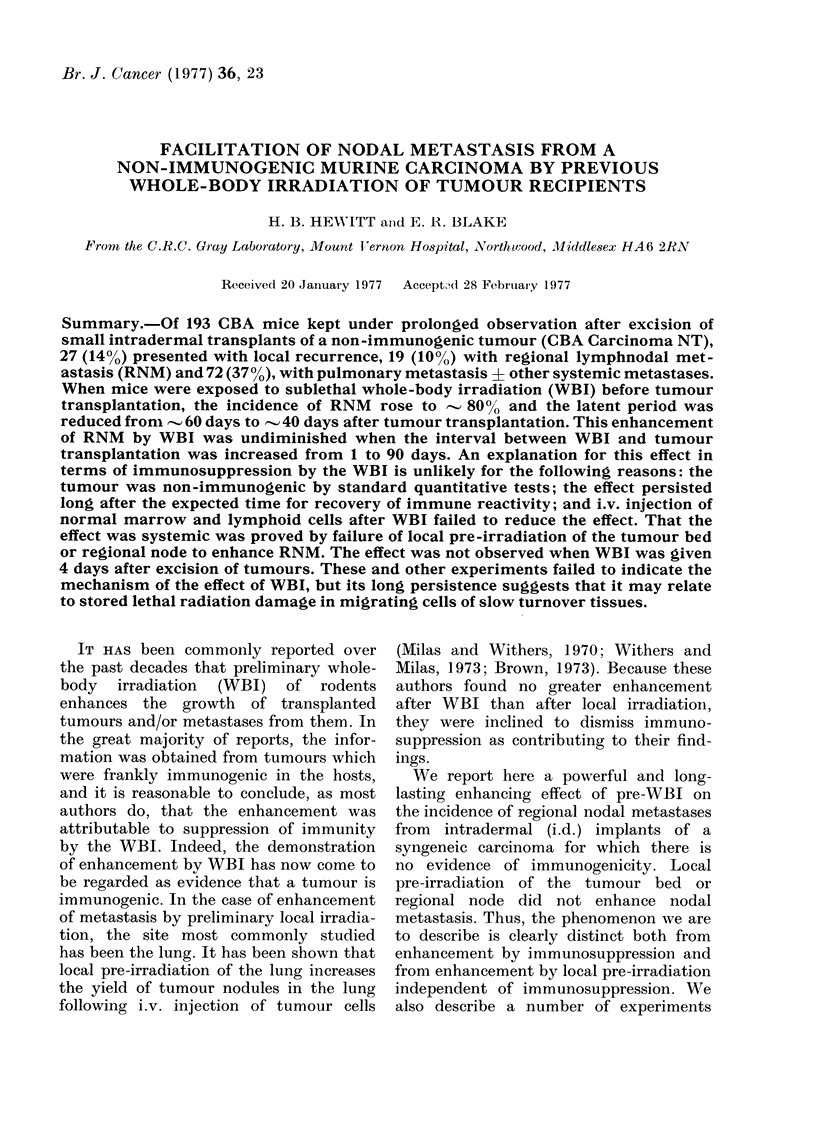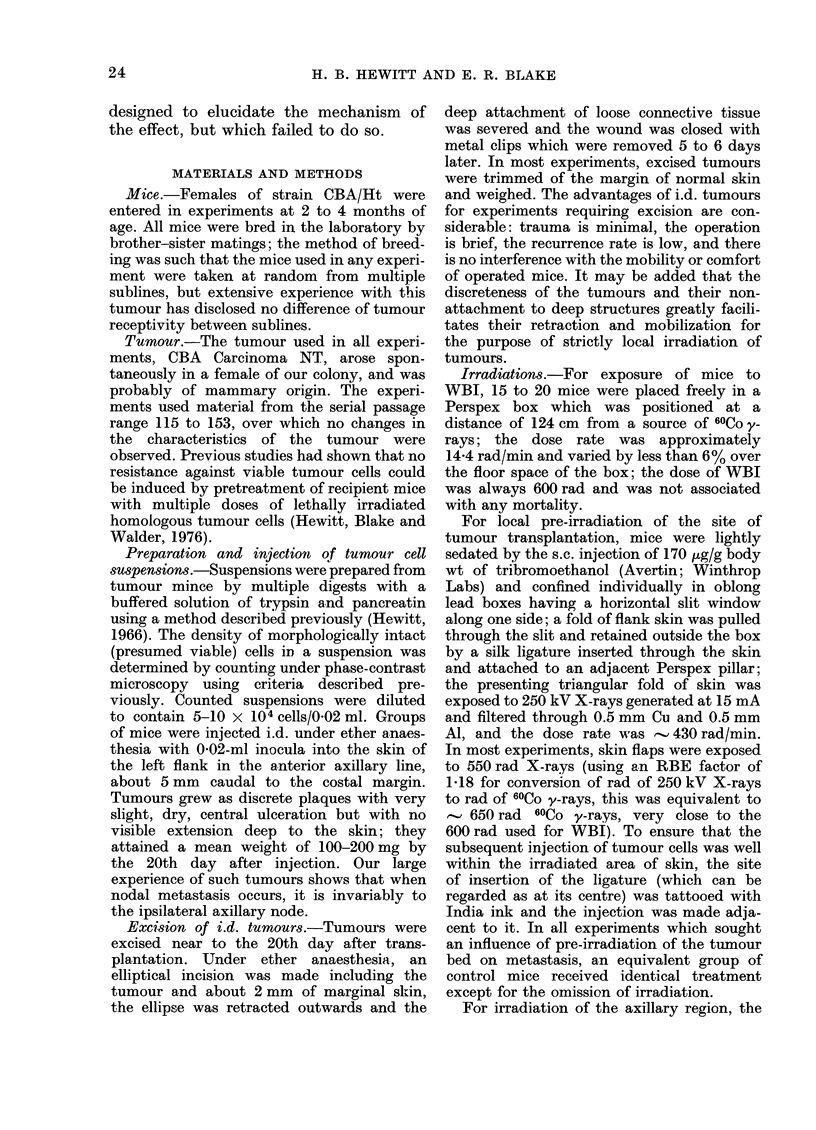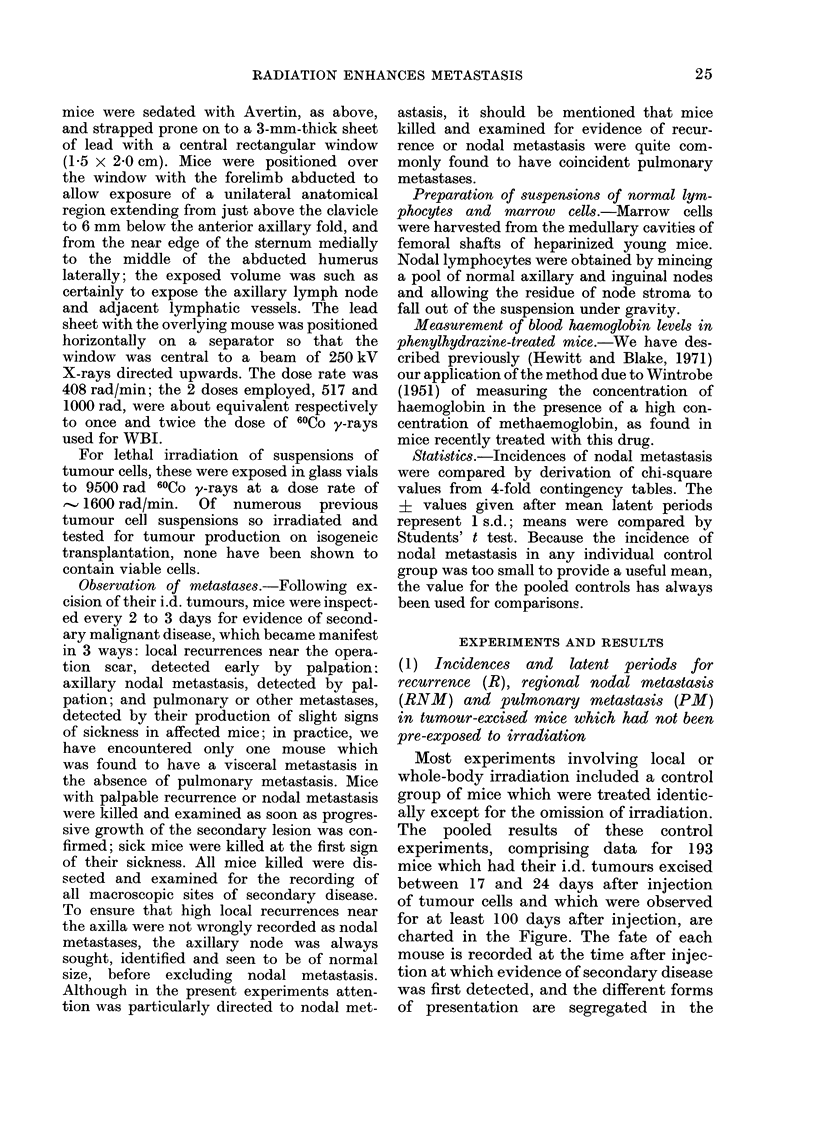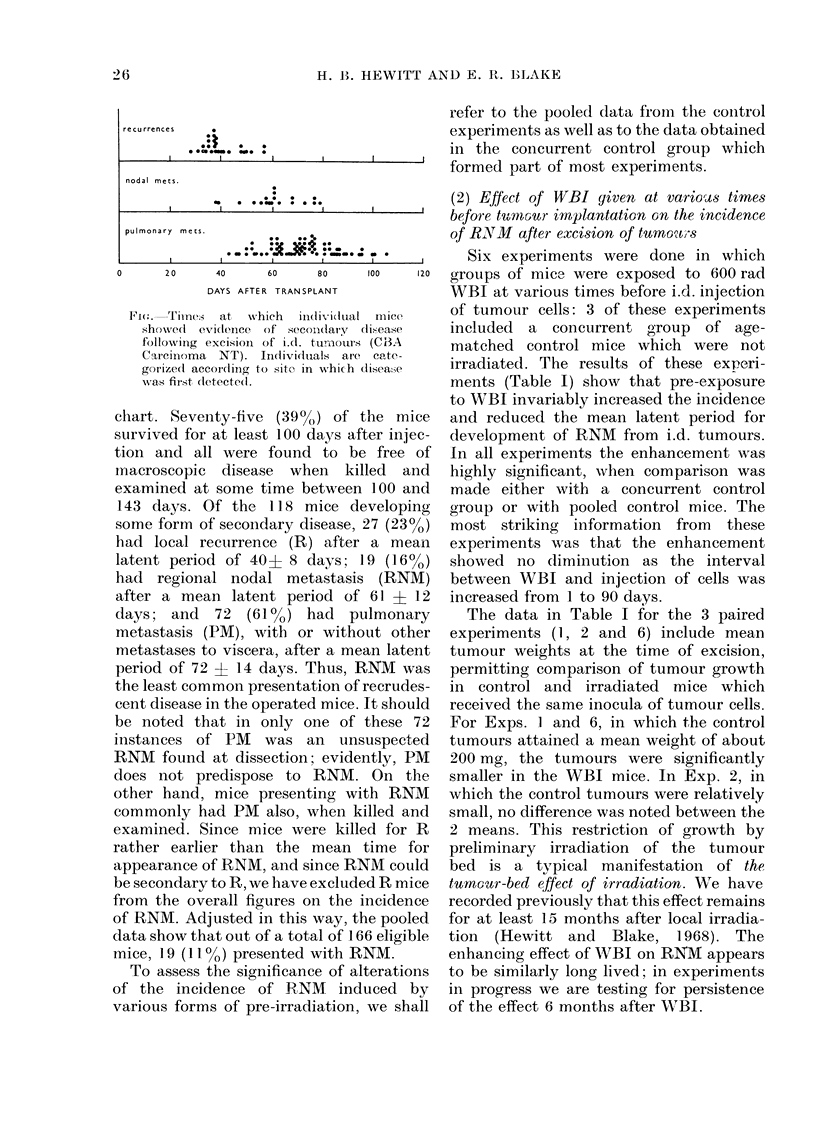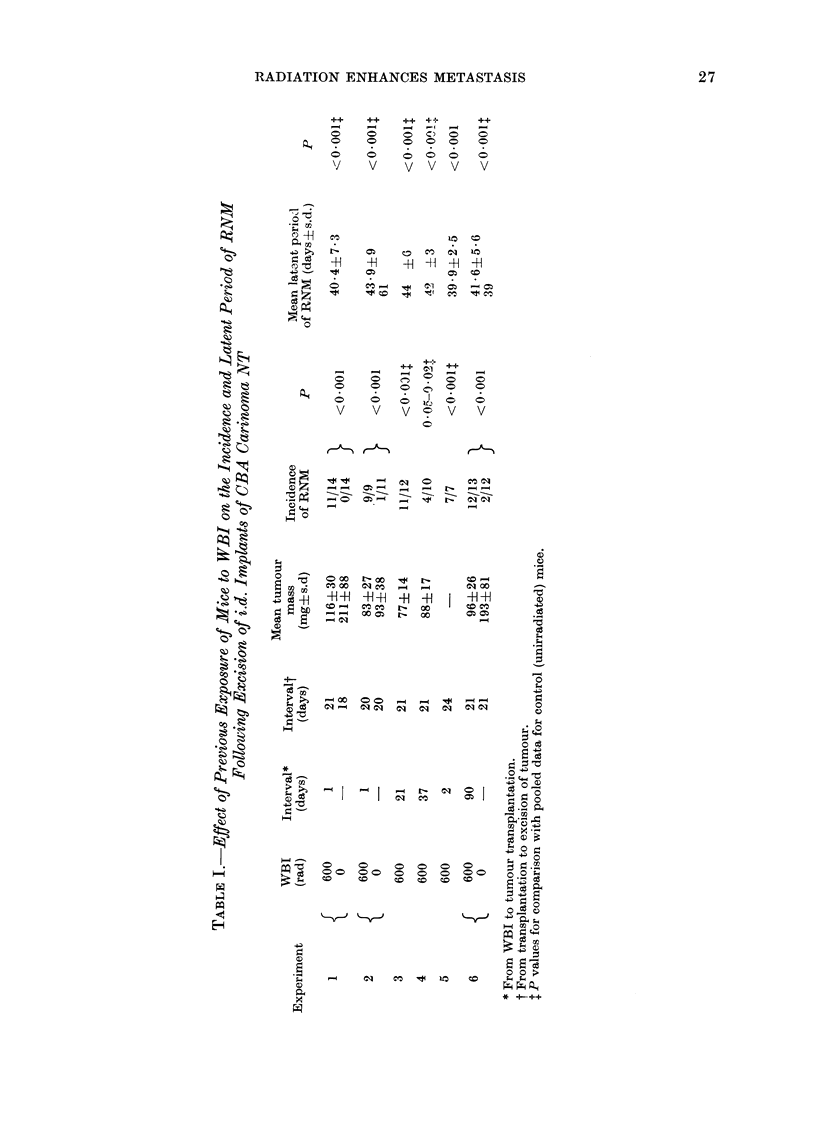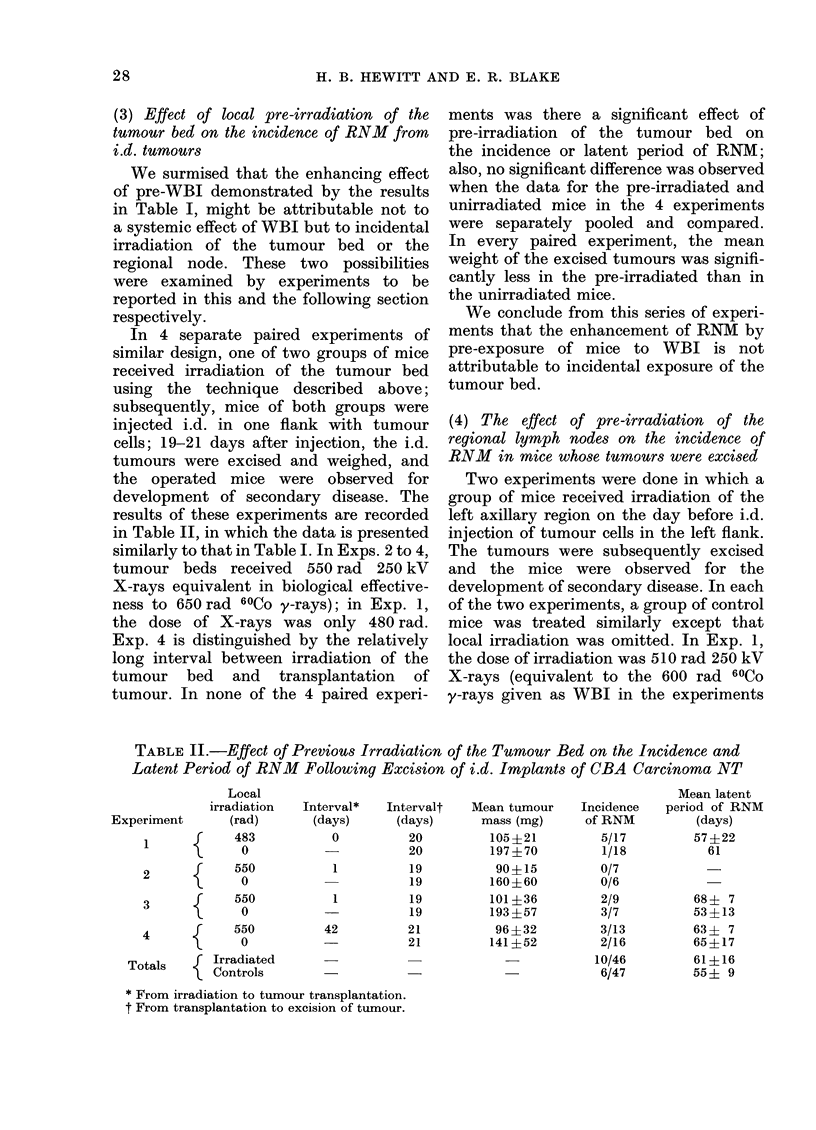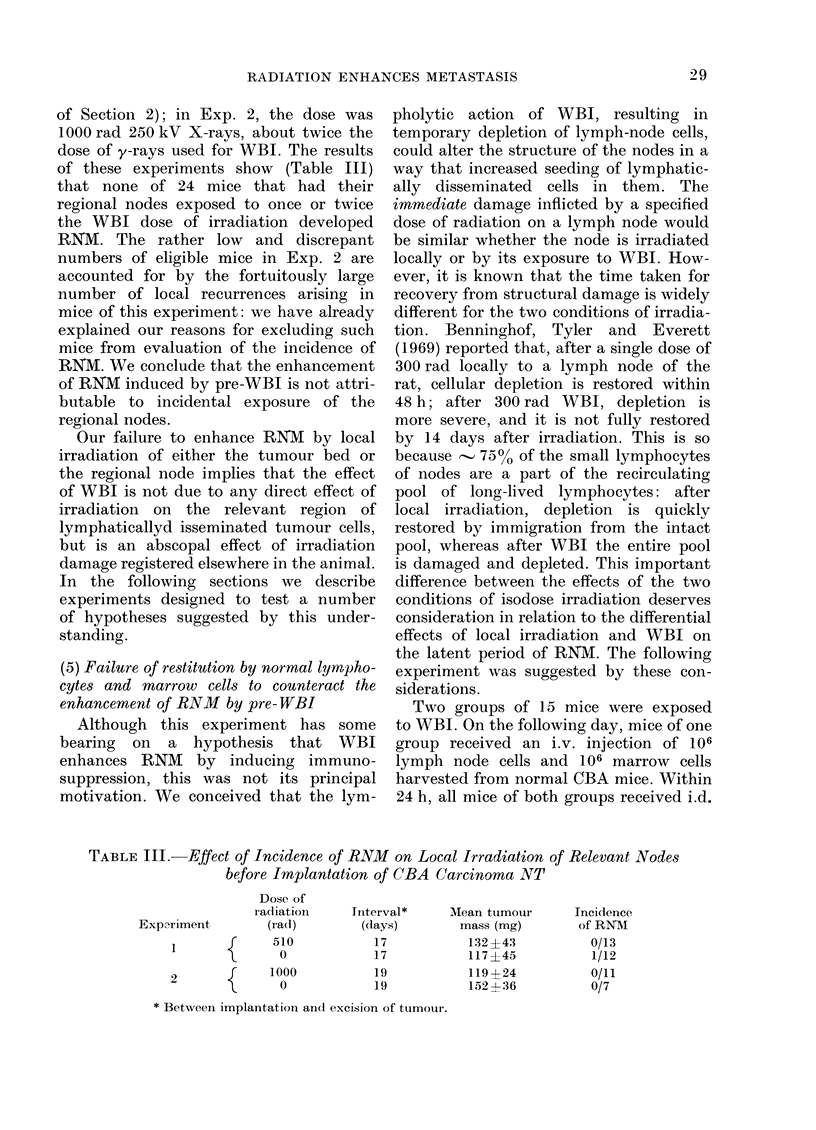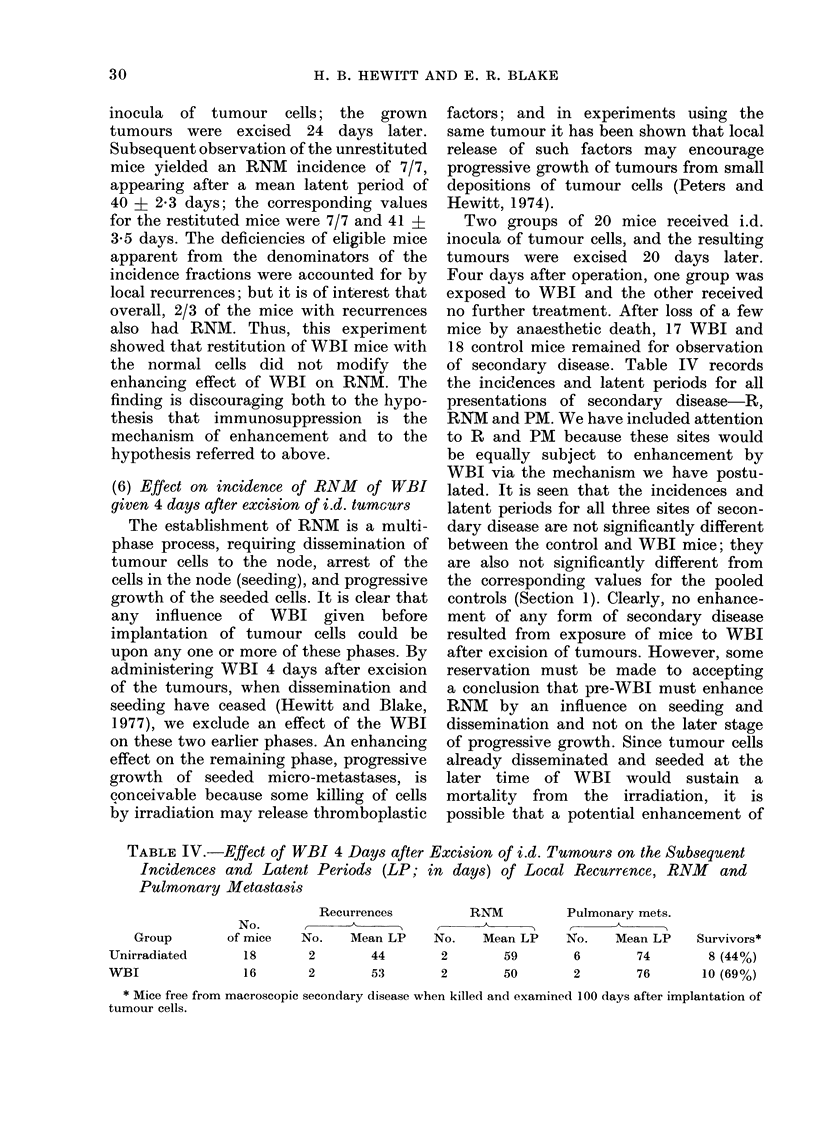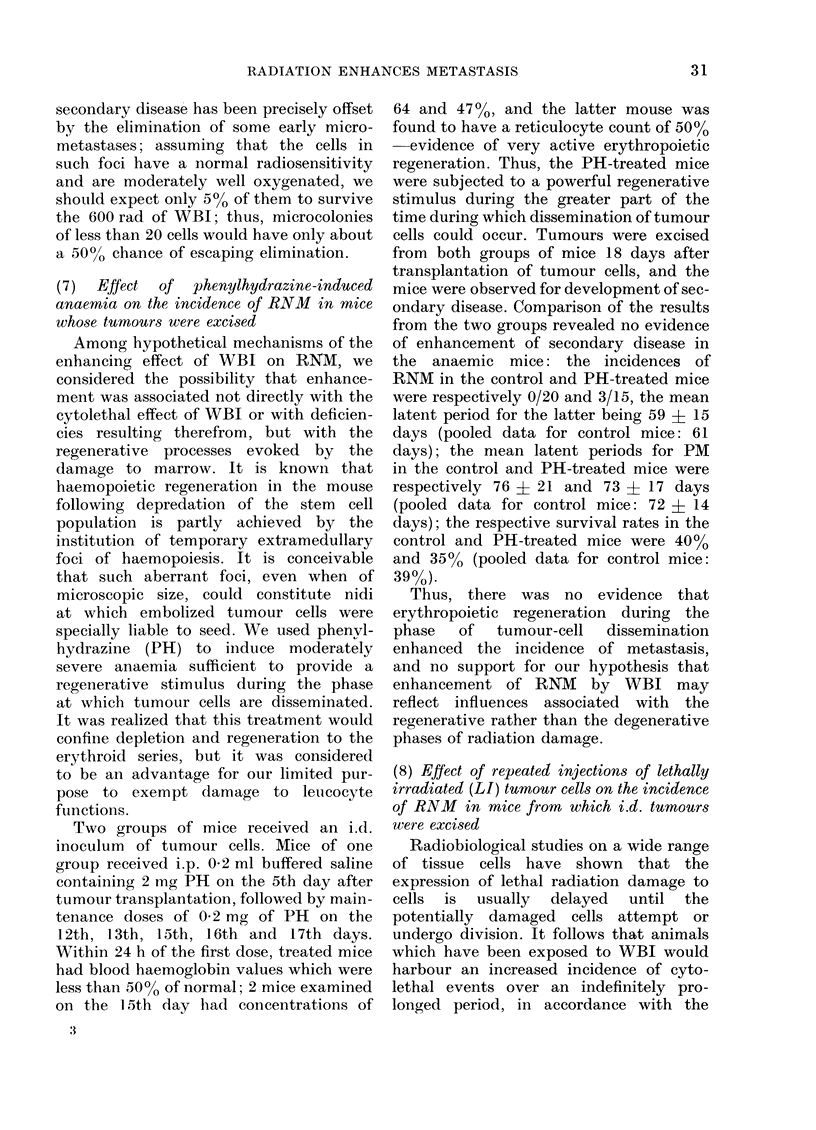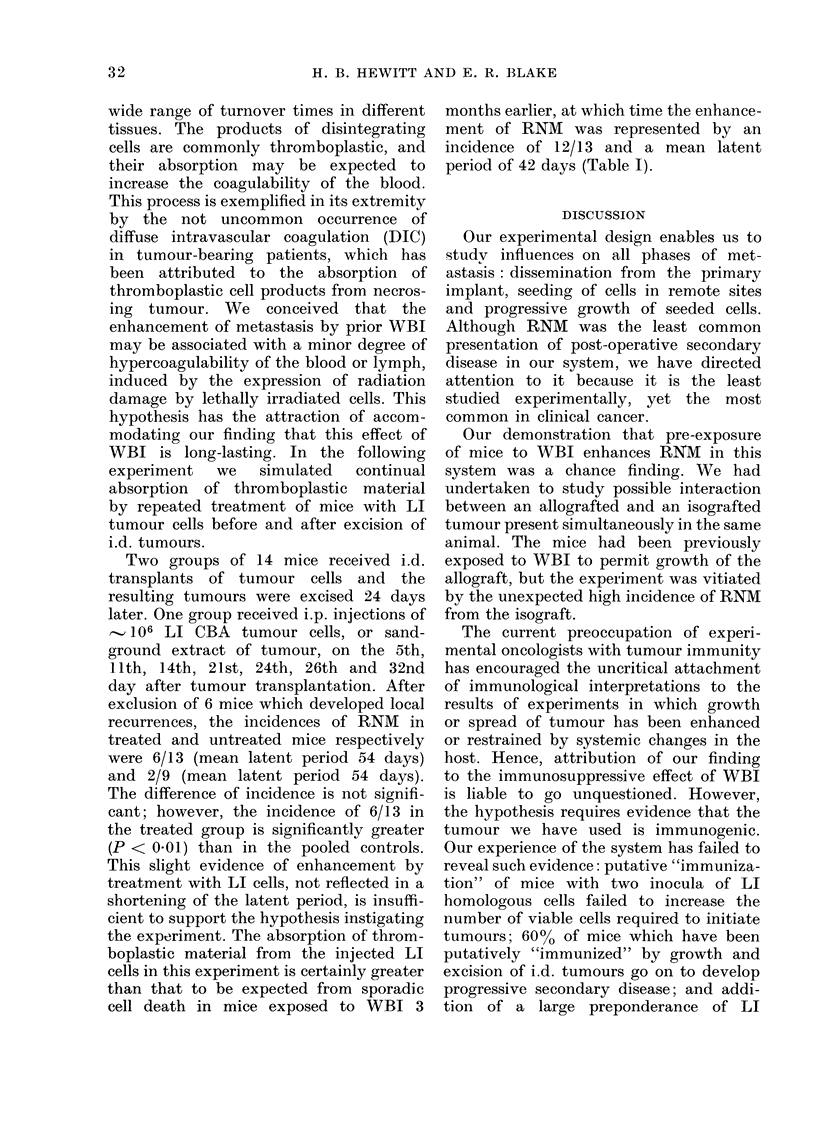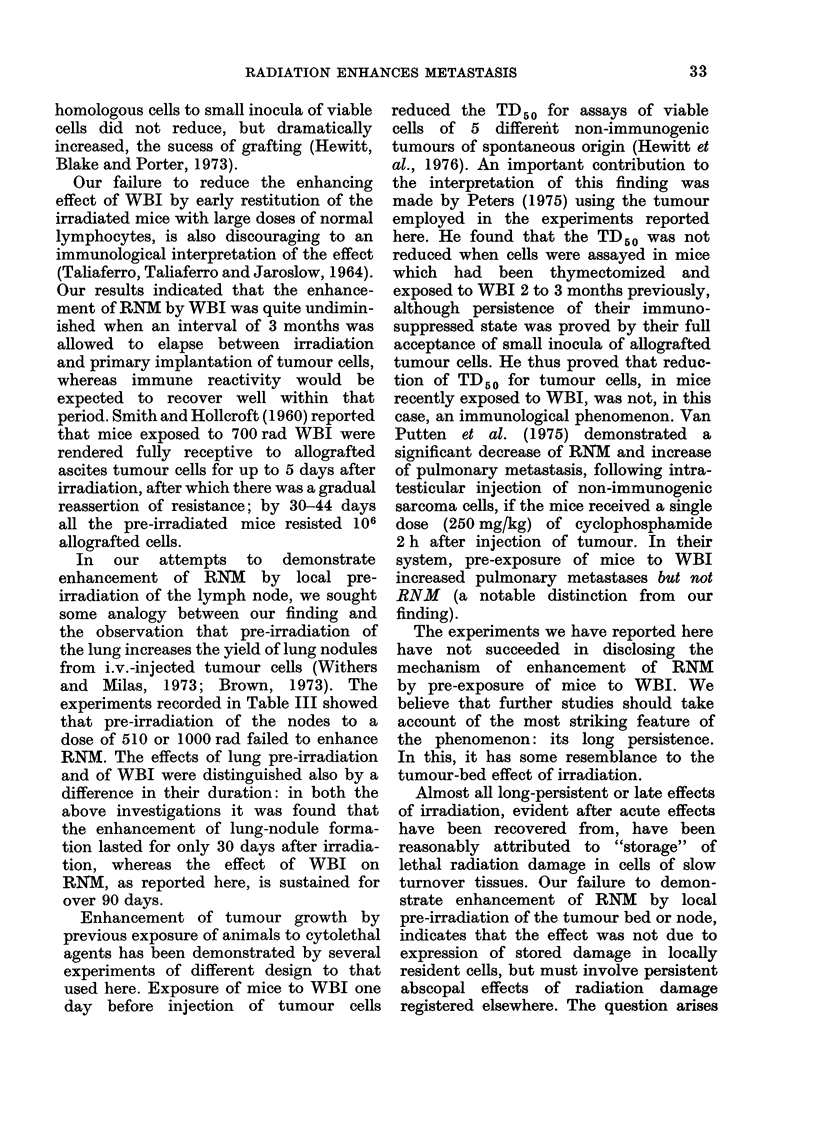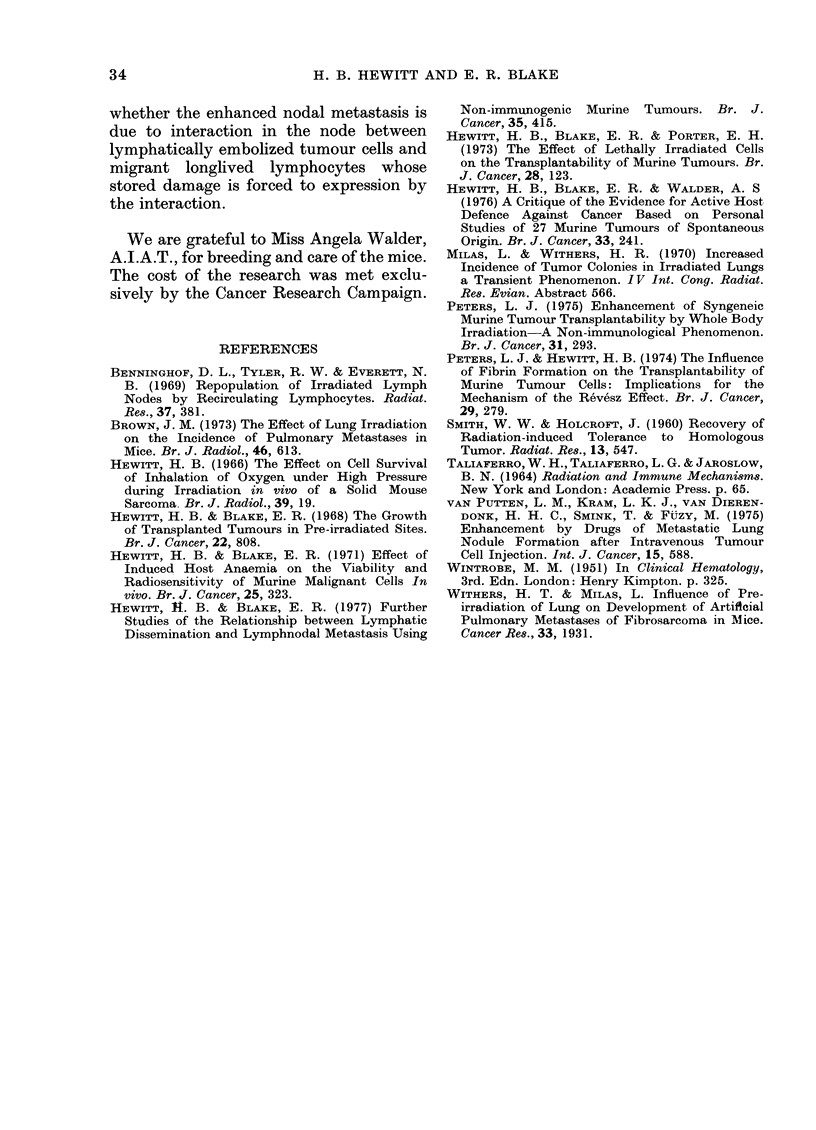# Facilitation of nodal metastasis from a non-immunogenic murine carcinoma by previous whole-body irradiation of tumour recipients.

**DOI:** 10.1038/bjc.1977.150

**Published:** 1977-07

**Authors:** H. B. Hewitt, E. R. Blake

## Abstract

Of 193 CBA mice kept under prolonged observation after excision of small intradermal transplants of a non-immunogenic tumour (CBA Carcinoma NT), 27 (14%) presented with local recurrence, 19 (10%) with regional lymphnodal metastasis (RNM) and 72 (37%), with pulmonary metastasis +/- other systemic metastases. When mice were exposed to sublethal whole-body irradiation (WBI) before tumour transplantation, the incidence of RNM rose to approximately 80% and the latent period was reduced from approximately 60 days to approximately 40 days after tumour transplantation. This enhancement of RNM by WBI was undiminished when the interval between WBI and tumour transplantation was increased from 1 to 90 days. An explanation for this effect in terms of immunosuppression by the WBI is unlikely for the following reasons: the tumour was non-immunogenic by standard quantitative tests; the effect persisted long after the expected time for recovery of immune reactivity; and i.v. injection of normal marrow and lymphoid cells after WBI failed to reduce the effect. That the effect was systemic was proved by failure of local pre-irradiation of the tumour bed or regional node to enhance RNM. The effect was not observed when WBI was given 4 days after excision of tumours. These and other experiments failed to indicate the mechanism of the effect of WBI, but its long persistence suggests that it may relate to stored lethal radiation damage in migrating cells of slow turnover tissues.


Br. J. Cancer (1977) 36, 23

### FACILITATION OF NODAL METASTASIS FROM A NON-IMMUNOGENIC MURINE CARCINOMA BY PREVIOUS WHOLE-BODY IRRADIATION OF TUMOUR RECIPIENTS

H. B. HEWITT and E. R. BLAKE

*From the C.R.C. Gray Laboratory, Mount Vernon Hospital, Northwood, Middlesex HA6 2RN*

Received 20 January 1977 Accepted 28 February 1977

**Summary.—Of 193 CBA mice kept under prolonged observation after excision of small intradermal transplants of a non-immunogenic tumour (CBA Carcinoma NT), 27 (14%) presented with local recurrence, 19 (10%) with regional lymphnodal metastasis (RNM) and 72 (37%), with pulmonary metastasis $\pm$ other systemic metastases. When mice were exposed to sublethal whole-body irradiation (WBI) before tumour transplantation, the incidence of RNM rose to $\sim$ 80% and the latent period was reduced from $\sim$60 days to $\sim$40 days after tumour transplantation. This enhancement of RNM by WBI was undiminished when the interval between WBI and tumour transplantation was increased from 1 to 90 days. An explanation for this effect in terms of immunosuppression by the WBI is unlikely for the following reasons: the tumour was non-immunogenic by standard quantitative tests; the effect persisted long after the expected time for recovery of immune reactivity; and i.v. injection of normal marrow and lymphoid cells after WBI failed to reduce the effect. That the effect was systemic was proved by failure of local pre-irradiation of the tumour bed or regional node to enhance RNM. The effect was not observed when WBI was given 4 days after excision of tumours. These and other experiments failed to indicate the mechanism of the effect of WBI, but its long persistence suggests that it may relate to stored lethal radiation damage in migrating cells of slow turnover tissues.**

It has been commonly reported over the past decades that preliminary whole-body irradiation (WBI) of rodents enhances the growth of transplanted tumours and/or metastases from them. In the great majority of reports, the information was obtained from tumours which were frankly immunogenic in the hosts, and it is reasonable to conclude, as most authors do, that the enhancement was attributable to suppression of immunity by the WBI. Indeed, the demonstration of enhancement by WBI has now come to be regarded as evidence that a tumour is immunogenic. In the case of enhancement of metastasis by preliminary local irradiation, the site most commonly studied has been the lung. It has been shown that local pre-irradiation of the lung increases the yield of tumour nodules in the lung following i.v. injection of tumour cells (Milas and Withers, 1970; Withers and Milas, 1973; Brown, 1973). Because these authors found no greater enhancement after WBI than after local irradiation, they were inclined to dismiss immunosuppression as contributing to their findings.

We report here a powerful and long-lasting enhancing effect of pre-WBI on the incidence of regional nodal metastases from intradermal (i.d.) implants of a syngeneic carcinoma for which there is no evidence of immunogenicity. Local pre-irradiation of the tumour bed or regional node did not enhance nodal metastasis. Thus, the phenomenon we are to describe is clearly distinct both from enhancement by immunosuppression and from enhancement by local pre-irradiation independent of immunosuppression. We also describe a number of experiments designed to elucidate the mechanism of the effect, but which failed to do so.

### MATERIALS AND METHODS

*Mice.*—Females of strain CBA/Ht were entered in experiments at 2 to 4 months of age. All mice were bred in the laboratory by brother–sister matings; the method of breeding was such that the mice used in any experiment were taken at random from multiple sublines, but extensive experience with this tumour has disclosed no difference of tumour receptivity between sublines.

*Tumour.*—The tumour used in all experiments, CBA Carcinoma NT, arose spontaneously in a female of our colony, and was probably of mammary origin. The experiments used material from the serial passage range 115 to 153, over which no changes in the characteristics of the tumour were observed. Previous studies had shown that no resistance against viable tumour cells could be induced by pretreatment of recipient mice with multiple doses of lethally irradiated homologous tumour cells (Hewitt, Blake and Walder, 1976).

*Preparation and injection of tumour cell suspensions.*—Suspensions were prepared from tumour mince by multiple digests with a buffered solution of trypsin and pancreatin using a method described previously (Hewitt, 1966). The density of morphologically intact (presumed viable) cells in a suspension was determined by counting under phase-contrast microscopy using criteria described previously. Counted suspensions were diluted to contain 5–10 $\times$ $10^4$ cells/0·02 ml. Groups of mice were injected i.d. under ether anaesthesia with 0·02-ml inocula into the skin of the left flank in the anterior axillary line, about 5 mm caudal to the costal margin. Tumours grew as discrete plaques with very slight, dry, central ulceration but with no visible extension deep to the skin; they attained a mean weight of 100–200 mg by the 20th day after injection. Our large experience of such tumours shows that when nodal metastasis occurs, it is invariably to the ipsilateral axillary node.

*Excision of i.d. tumours.*—Tumours were excised near to the 20th day after transplantation. Under ether anaesthesia, an elliptical incision was made including the tumour and about 2 mm of marginal skin, the ellipse was retracted outwards and the deep attachment of loose connective tissue was severed and the wound was closed with metal clips which were removed 5 to 6 days later. In most experiments, excised tumours were trimmed of the margin of normal skin and weighed. The advantages of i.d. tumours for experiments requiring excision are considerable: trauma is minimal, the operation is brief, the recurrence rate is low, and there is no interference with the mobility or comfort of operated mice. It may be added that the discreteness of the tumours and their non-attachment to deep structures greatly facilitates their retraction and mobilization for the purpose of strictly local irradiation of tumours.

*Irradiations.*—For exposure of mice to WBI, 15 to 20 mice were placed freely in a Perspex box which was positioned at a distance of 124 cm from a source of $^{60}$Co $\gamma$-rays; the dose rate was approximately 14·4 rad/min and varied by less than 6% over the floor space of the box; the dose of WBI was always 600 rad and was not associated with any mortality.

For local pre-irradiation of the site of tumour transplantation, mice were lightly sedated by the s.c. injection of 170 $\mu$g/g body wt of tribromoethanol (Avertin; Winthrop Labs) and confined individually in oblong lead boxes having a horizontal slit window along one side; a fold of flank skin was pulled through the slit and retained outside the box by a silk ligature inserted through the skin and attached to an adjacent Perspex pillar; the presenting triangular fold of skin was exposed to 250 kV X-rays generated at 15 mA and filtered through 0.5 mm Cu and 0.5 mm Al, and the dose rate was $\sim$430 rad/min. In most experiments, skin flaps were exposed to 550 rad X-rays (using an RBE factor of 1·18 for conversion of rad of 250 kV X-rays to rad of $^{60}$Co $\gamma$-rays, this was equivalent to $\sim$ 650 rad $^{60}$Co $\gamma$-rays, very close to the 600 rad used for WBI). To ensure that the subsequent injection of tumour cells was well within the irradiated area of skin, the site of insertion of the ligature (which can be regarded as at its centre) was tattooed with India ink and the injection was made adjacent to it. In all experiments which sought an influence of pre-irradiation of the tumour bed on metastasis, an equivalent group of control mice received identical treatment except for the omission of irradiation.

For irradiation of the axillary region, the mice were sedated with Avertin, as above, and strapped prone on to a 3-mm-thick sheet of lead with a central rectangular window (1·5 × 2·0 cm). Mice were positioned over the window with the forelimb abducted to allow exposure of a unilateral anatomical region extending from just above the clavicle to 6 mm below the anterior axillary fold, and from the near edge of the sternum medially to the middle of the abducted humerus laterally; the exposed volume was such as certainly to expose the axillary lymph node and adjacent lymphatic vessels. The lead sheet with the overlying mouse was positioned horizontally on a separator so that the window was central to a beam of 250 kV X-rays directed upwards. The dose rate was 408 rad/min; the 2 doses employed, 517 and 1000 rad, were about equivalent respectively to once and twice the dose of $^{60}$Co $\gamma$-rays used for WBI.

For lethal irradiation of suspensions of tumour cells, these were exposed in glass vials to 9500 rad $^{60}$Co $\gamma$-rays at a dose rate of $\sim$ 1600 rad/min. Of numerous previous tumour cell suspensions so irradiated and tested for tumour production on isogeneic transplantation, none have been shown to contain viable cells.

*Observation of metastases.*—Following excision of their i.d. tumours, mice were inspected every 2 to 3 days for evidence of secondary malignant disease, which became manifest in 3 ways: local recurrences near the operation scar, detected early by palpation; axillary nodal metastasis, detected by palpation; and pulmonary or other metastases, detected by their production of slight signs of sickness in affected mice; in practice, we have encountered only one mouse which was found to have a visceral metastasis in the absence of pulmonary metastasis. Mice with palpable recurrence or nodal metastasis were killed and examined as soon as progressive growth of the secondary lesion was confirmed; sick mice were killed at the first sign of their sickness. All mice killed were dissected and examined for the recording of all macroscopic sites of secondary disease. To ensure that high local recurrences near the axilla were not wrongly recorded as nodal metastases, the axillary node was always sought, identified and seen to be of normal size, before excluding nodal metastasis. Although in the present experiments attention was particularly directed to nodal metastasis, it should be mentioned that mice killed and examined for evidence of recurrence or nodal metastasis were quite commonly found to have coincident pulmonary metastases.

*Preparation of suspensions of normal lymphocytes and marrow cells.*—Marrow cells were harvested from the medullary cavities of femoral shafts of heparinized young mice. Nodal lymphocytes were obtained by mincing a pool of normal axillary and inguinal nodes and allowing the residue of node stroma to fall out of the suspension under gravity.

*Measurement of blood haemoglobin levels in phenylhydrazine-treated mice.*—We have described previously (Hewitt and Blake, 1971) our application of the method due to Wintrobe (1951) of measuring the concentration of haemoglobin in the presence of a high concentration of methaemoglobin, as found in mice recently treated with this drug.

*Statistics.*—Incidences of nodal metastasis were compared by derivation of chi-square values from 4-fold contingency tables. The ± values given after mean latent periods represent 1 s.d.; means were compared by Students' *t* test. Because the incidence of nodal metastasis in any individual control group was too small to provide a useful mean, the value for the pooled controls has always been used for comparisons.

### EXPERIMENTS AND RESULTS

### (1) *Incidences and latent periods for recurrence (R), regional nodal metastasis (RNM) and pulmonary metastasis (PM) in tumour-excised mice which had not been pre-exposed to irradiation*

Most experiments involving local or whole-body irradiation included a control group of mice which were treated identically except for the omission of irradiation. The pooled results of these control experiments, comprising data for 193 mice which had their i.d. tumours excised between 17 and 24 days after injection of tumour cells and which were observed for at least 100 days after injection, are charted in the Figure. The fate of each mouse is recorded at the time after injection at which evidence of secondary disease was first detected, and the different forms of presentation are segregated in the

FIG.—Times at which individual mice showed evidence of secondary disease following excision of i.d. tumours (CBA Carcinoma NT). Individuals are categorized according to site in which disease was first detected.

chart. Seventy-five (39%) of the mice survived for at least 100 days after injection and all were found to be free of macroscopic disease when killed and examined at some time between 100 and 143 days. Of the 118 mice developing some form of secondary disease, 27 (23%) had local recurrence (R) after a mean latent period of 40 $\pm$ 8 days; 19 (16%) had regional nodal metastasis (RNM) after a mean latent period of 61 $\pm$ 12 days; and 72 (61%) had pulmonary metastasis (PM), with or without other metastases to viscera, after a mean latent period of 72 $\pm$ 14 days. Thus, RNM was the least common presentation of recrudescent disease in the operated mice. It should be noted that in only one of these 72 instances of PM was an unsuspected RNM found at dissection; evidently, PM does not predispose to RNM. On the other hand, mice presenting with RNM commonly had PM also, when killed and examined. Since mice were killed for R rather earlier than the mean time for appearance of RNM, and since RNM could be secondary to R, we have excluded R mice from the overall figures on the incidence of RNM. Adjusted in this way, the pooled data show that out of a total of 166 eligible mice, 19 (11%) presented with RNM.

To assess the significance of alterations of the incidence of RNM induced by various forms of pre-irradiation, we shall refer to the pooled data from the control experiments as well as to the data obtained in the concurrent control group which formed part of most experiments.

(2) *Effect of WBI given at various times before tumour implantation on the incidence of RNM after excision of tumours*

Six experiments were done in which groups of mice were exposed to 600 rad WBI at various times before i.d. injection of tumour cells: 3 of these experiments included a concurrent group of age-matched control mice which were not irradiated. The results of these experiments (Table I) show that pre-exposure to WBI invariably increased the incidence and reduced the mean latent period for development of RNM from i.d. tumours. In all experiments the enhancement was highly significant, when comparison was made either with a concurrent control group or with pooled control mice. The most striking information from these experiments was that the enhancement showed no diminution as the interval between WBI and injection of cells was increased from 1 to 90 days.

The data in Table I for the 3 paired experiments (1, 2 and 6) include mean tumour weights at the time of excision, permitting comparison of tumour growth in control and irradiated mice which received the same inocula of tumour cells. For Exps. 1 and 6, in which the control tumours attained a mean weight of about 200 mg, the tumours were significantly smaller in the WBI mice. In Exp. 2, in which the control tumours were relatively small, no difference was noted between the 2 means. This restriction of growth by preliminary irradiation of the tumour bed is a typical manifestation of *the tumour-bed effect of irradiation*. We have recorded previously that this effect remains for at least 15 months after local irradiation (Hewitt and Blake, 1968). The enhancing effect of WBI on RNM appears to be similarly long lived; in experiments in progress we are testing for persistence of the effect 6 months after WBI.

TABLE I.—*Effect of Previous Exposure of Mice to WBI on the Incidence and Latent Period of RNM Following Excision of i.d. Implants of CBA Carinoma NT*

| Experiment | WBI (rad) | Interval* (days) | Interval† (days) | Mean tumour mass (mg±s.d) | Incidence of RNM | P | Mean latent period of RNM (days±s.d.) | P |
|---|---|---|---|---|---|---|---|---|
| 1 | 600 | 1 | 21 | 116±30 | 11/14 | <0·001 | 40·4±7·3 | <0·001‡ |
| | 0 | — | 18 | 211±88 | 0/14 | | | |
| 2 | 600 | 1 | 20 | 83±27 | 9/9 | <0·001 | 43·9±9 | <0·001‡ |
| | 0 | — | 20 | 93±38 | 1/11 | | 61 | |
| 3 | 600 | 21 | 21 | 77±14 | 11/12 | <0·001‡ | 44 ±6 | <0·001‡ |
| 4 | 600 | 37 | 21 | 88±17 | 4/10 | 0·05–0·02‡ | 42 ±3 | <0·001‡ |
| 5 | 600 | 2 | 24 | — | 7/7 | <0·001‡ | 39·9±2·5 | <0·001 |
| 6 | 600 | 90 | 21 | 96±26 | 12/13 | <0·001 | 41·6±5·6 | <0·001‡ |
| | 0 | — | 21 | 193±81 | 2/12 | | 39 | |

* From WBI to tumour transplantation.

† From transplantation to excision of tumour.

‡ *P* values for comparison with pooled data for control (unirradiated) mice.

(3) *Effect of local pre-irradiation of the tumour bed on the incidence of RNM from i.d. tumours*

We surmised that the enhancing effect of pre-WBI demonstrated by the results in Table I, might be attributable not to a systemic effect of WBI but to incidental irradiation of the tumour bed or the regional node. These two possibilities were examined by experiments to be reported in this and the following section respectively.

In 4 separate paired experiments of similar design, one of two groups of mice received irradiation of the tumour bed using the technique described above; subsequently, mice of both groups were injected i.d. in one flank with tumour cells; 19–21 days after injection, the i.d. tumours were excised and weighed, and the operated mice were observed for development of secondary disease. The results of these experiments are recorded in Table II, in which the data is presented similarly to that in Table I. In Exps. 2 to 4, tumour beds received 550 rad 250 kV X-rays equivalent in biological effectiveness to 650 rad $^{60}Co$ $\gamma$-rays); in Exp. 1, the dose of X-rays was only 480 rad. Exp. 4 is distinguished by the relatively long interval between irradiation of the tumour bed and transplantation of tumour. In none of the 4 paired experiments was there a significant effect of pre-irradiation of the tumour bed on the incidence or latent period of RNM; also, no significant difference was observed when the data for the pre-irradiated and unirradiated mice in the 4 experiments were separately pooled and compared. In every paired experiment, the mean weight of the excised tumours was significantly less in the pre-irradiated than in the unirradiated mice.

We conclude from this series of experiments that the enhancement of RNM by pre-exposure of mice to WBI is not attributable to incidental exposure of the tumour bed.

(4) *The effect of pre-irradiation of the regional lymph nodes on the incidence of RNM in mice whose tumours were excised*

Two experiments were done in which a group of mice received irradiation of the left axillary region on the day before i.d. injection of tumour cells in the left flank. The tumours were subsequently excised and the mice were observed for the development of secondary disease. In each of the two experiments, a group of control mice was treated similarly except that local irradiation was omitted. In Exp. 1, the dose of irradiation was 510 rad 250 kV X-rays (equivalent to the 600 rad $^{60}Co$ $\gamma$-rays given as WBI in the experiments

TABLE II.—*Effect of Previous Irradiation of the Tumour Bed on the Incidence and Latent Period of RNM Following Excision of i.d. Implants of CBA Carcinoma NT*

| Experiment | Local irradiation (rad) | Interval* (days) | Interval† (days) | Mean tumour mass (mg) | Incidence of RNM | Mean latent period of RNM (days) |
|---|---|---|---|---|---|---|
| 1 | 483 | 0 | 20 | 105±21 | 5/17 | 57±22 |
| | 0 | — | 20 | 197±70 | 1/18 | 61 |
| 2 | 550 | 1 | 19 | 90±15 | 0/7 | — |
| | 0 | — | 19 | 160±60 | 0/6 | — |
| 3 | 550 | 1 | 19 | 101±36 | 2/9 | 68± 7 |
| | 0 | — | 19 | 193±57 | 3/7 | 53±13 |
| 4 | 550 | 42 | 21 | 96±32 | 3/13 | 63± 7 |
| | 0 | — | 21 | 141±52 | 2/16 | 65±17 |
| Totals | Irradiated | — | — | — | 10/46 | 61±16 |
| | Controls | — | — | — | 6/47 | 55± 9 |

* From irradiation to tumour transplantation.
† From transplantation to excision of tumour.

of Section 2); in Exp. 2, the dose was 1000 rad 250 kV X-rays, about twice the dose of $\gamma$-rays used for WBI. The results of these experiments show (Table III) that none of 24 mice that had their regional nodes exposed to once or twice the WBI dose of irradiation developed RNM. The rather low and discrepant numbers of eligible mice in Exp. 2 are accounted for by the fortuitously large number of local recurrences arising in mice of this experiment: we have already explained our reasons for excluding such mice from evaluation of the incidence of RNM. We conclude that the enhancement of RNM induced by pre-WBI is not attributable to incidental exposure of the regional nodes.

Our failure to enhance RNM by local irradiation of either the tumour bed or the regional node implies that the effect of WBI is not due to any direct effect of irradiation on the relevant region of lymphaticallyd isseminated tumour cells, but is an abscopal effect of irradiation damage registered elsewhere in the animal. In the following sections we describe experiments designed to test a number of hypotheses suggested by this understanding.

(5) *Failure of restitution by normal lymphocytes and marrow cells to counteract the enhancement of RNM by pre-WBI*

Although this experiment has some bearing on a hypothesis that WBI enhances RNM by inducing immunosuppression, this was not its principal motivation. We conceived that the lympholytic action of WBI, resulting in temporary depletion of lymph-node cells, could alter the structure of the nodes in a way that increased seeding of lymphatically disseminated cells in them. The *immediate* damage inflicted by a specified dose of radiation on a lymph node would be similar whether the node is irradiated locally or by its exposure to WBI. However, it is known that the time taken for recovery from structural damage is widely different for the two conditions of irradiation. Benninghof, Tyler and Everett (1969) reported that, after a single dose of 300 rad locally to a lymph node of the rat, cellular depletion is restored within 48 h; after 300 rad WBI, depletion is more severe, and it is not fully restored by 14 days after irradiation. This is so because $\sim 75\%$ of the small lymphocytes of nodes are a part of the recirculating pool of long-lived lymphocytes: after local irradiation, depletion is quickly restored by immigration from the intact pool, whereas after WBI the entire pool is damaged and depleted. This important difference between the effects of the two conditions of isodose irradiation deserves consideration in relation to the differential effects of local irradiation and WBI on the latent period of RNM. The following experiment was suggested by these considerations.

Two groups of 15 mice were exposed to WBI. On the following day, mice of one group received an i.v. injection of $10^6$ lymph node cells and $10^6$ marrow cells harvested from normal CBA mice. Within 24 h, all mice of both groups received i.d.

TABLE III.—*Effect of Incidence of RNM on Local Irradiation of Relevant Nodes before Implantation of CBA Carcinoma NT*

| Experiment | Dose of radiation (rad) | Interval* (days) | Mean tumour mass (mg) | Incidence of RNM |
|---|---|---|---|---|
| 1 | 510 | 17 | 132±43 | 0/13 |
| | 0 | 17 | 117±45 | 1/12 |
| 2 | 1000 | 19 | 119±24 | 0/11 |
| | 0 | 19 | 152±36 | 0/7 |

* Between implantation and excision of tumour.

inocula of tumour cells; the grown tumours were excised 24 days later. Subsequent observation of the unrestituted mice yielded an RNM incidence of 7/7, appearing after a mean latent period of $40 \pm 2{\cdot}3$ days; the corresponding values for the restituted mice were 7/7 and $41 \pm 3{\cdot}5$ days. The deficiencies of eligible mice apparent from the denominators of the incidence fractions were accounted for by local recurrences; but it is of interest that overall, 2/3 of the mice with recurrences also had RNM. Thus, this experiment showed that restitution of WBI mice with the normal cells did not modify the enhancing effect of WBI on RNM. The finding is discouraging both to the hypothesis that immunosuppression is the mechanism of enhancement and to the hypothesis referred to above.

(6) *Effect on incidence of RNM of WBI given* 4 *days after excision of i.d. tumours*

The establishment of RNM is a multiphase process, requiring dissemination of tumour cells to the node, arrest of the cells in the node (seeding), and progressive growth of the seeded cells. It is clear that any influence of WBI given before implantation of tumour cells could be upon any one or more of these phases. By administering WBI 4 days after excision of the tumours, when dissemination and seeding have ceased (Hewitt and Blake, 1977), we exclude an effect of the WBI on these two earlier phases. An enhancing effect on the remaining phase, progressive growth of seeded micro-metastases, is conceivable because some killing of cells by irradiation may release thromboplastic factors; and in experiments using the same tumour it has been shown that local release of such factors may encourage progressive growth of tumours from small depositions of tumour cells (Peters and Hewitt, 1974).

Two groups of 20 mice received i.d. inocula of tumour cells, and the resulting tumours were excised 20 days later. Four days after operation, one group was exposed to WBI and the other received no further treatment. After loss of a few mice by anaesthetic death, 17 WBI and 18 control mice remained for observation of secondary disease. Table IV records the incidences and latent periods for all presentations of secondary disease—R, RNM and PM. We have included attention to R and PM because these sites would be equally subject to enhancement by WBI via the mechanism we have postulated. It is seen that the incidences and latent periods for all three sites of secondary disease are not significantly different between the control and WBI mice; they are also not significantly different from the corresponding values for the pooled controls (Section 1). Clearly, no enhancement of any form of secondary disease resulted from exposure of mice to WBI after excision of tumours. However, some reservation must be made to accepting a conclusion that pre-WBI must enhance RNM by an influence on seeding and dissemination and not on the later stage of progressive growth. Since tumour cells already disseminated and seeded at the later time of WBI would sustain a mortality from the irradiation, it is possible that a potential enhancement of

TABLE IV.—*Effect of WBI* 4 *Days after Excision of i.d. Tumours on the Subsequent Incidences and Latent Periods* (*LP; in days*) *of Local Recurrence, RNM and Pulmonary Metastasis*

| Group | No. of mice | Recurrences | | RNM | | Pulmonary mets. | | Survivors* |
|---|---|---|---|---|---|---|---|---|
| | | No. | Mean LP | No. | Mean LP | No. | Mean LP | |
| Unirradiated | 18 | 2 | 44 | 2 | 59 | 6 | 74 | 8 (44%) |
| WBI | 16 | 2 | 53 | 2 | 50 | 2 | 76 | 10 (69%) |

* Mice free from macroscopic secondary disease when killed and examined 100 days after implantation of tumour cells.

secondary disease has been precisely offset by the elimination of some early micrometastases; assuming that the cells in such foci have a normal radiosensitivity and are moderately well oxygenated, we should expect only 5% of them to survive the 600 rad of WBI; thus, microcolonies of less than 20 cells would have only about a 50% chance of escaping elimination.

(7) *Effect of phenylhydrazine-induced anaemia on the incidence of RNM in mice whose tumours were excised*

Among hypothetical mechanisms of the enhancing effect of WBI on RNM, we considered the possibility that enhancement was associated not directly with the cytolethal effect of WBI or with deficiencies resulting therefrom, but with the regenerative processes evoked by the damage to marrow. It is known that haemopoietic regeneration in the mouse following depredation of the stem cell population is partly achieved by the institution of temporary extramedullary foci of haemopoiesis. It is conceivable that such aberrant foci, even when of microscopic size, could constitute nidi at which embolized tumour cells were specially liable to seed. We used phenylhydrazine (PH) to induce moderately severe anaemia sufficient to provide a regenerative stimulus during the phase at which tumour cells are disseminated. It was realized that this treatment would confine depletion and regeneration to the erythroid series, but it was considered to be an advantage for our limited purpose to exempt damage to leucocyte functions.

Two groups of mice received an i.d. inoculum of tumour cells. Mice of one group received i.p. 0·2 ml buffered saline containing 2 mg PH on the 5th day after tumour transplantation, followed by maintenance doses of 0·2 mg of PH on the 12th, 13th, 15th, 16th and 17th days. Within 24 h of the first dose, treated mice had blood haemoglobin values which were less than 50% of normal; 2 mice examined on the 15th day had concentrations of 64 and 47%, and the latter mouse was found to have a reticulocyte count of 50% —evidence of very active erythropoietic regeneration. Thus, the PH-treated mice were subjected to a powerful regenerative stimulus during the greater part of the time during which dissemination of tumour cells could occur. Tumours were excised from both groups of mice 18 days after transplantation of tumour cells, and the mice were observed for development of secondary disease. Comparison of the results from the two groups revealed no evidence of enhancement of secondary disease in the anaemic mice: the incidences of RNM in the control and PH-treated mice were respectively 0/20 and 3/15, the mean latent period for the latter being 59 ± 15 days (pooled data for control mice: 61 days); the mean latent periods for PM in the control and PH-treated mice were respectively 76 ± 21 and 73 ± 17 days (pooled data for control mice: 72 ± 14 days); the respective survival rates in the control and PH-treated mice were 40% and 35% (pooled data for control mice: 39%).

Thus, there was no evidence that erythropoietic regeneration during the phase of tumour-cell dissemination enhanced the incidence of metastasis, and no support for our hypothesis that enhancement of RNM by WBI may reflect influences associated with the regenerative rather than the degenerative phases of radiation damage.

(8) *Effect of repeated injections of lethally irradiated (LI) tumour cells on the incidence of RNM in mice from which i.d. tumours were excised*

Radiobiological studies on a wide range of tissue cells have shown that the expression of lethal radiation damage to cells is usually delayed until the potentially damaged cells attempt or undergo division. It follows that animals which have been exposed to WBI would harbour an increased incidence of cytolethal events over an indefinitely prolonged period, in accordance with the wide range of turnover times in different tissues. The products of disintegrating cells are commonly thromboplastic, and their absorption may be expected to increase the coagulability of the blood. This process is exemplified in its extremity by the not uncommon occurrence of diffuse intravascular coagulation (DIC) in tumour-bearing patients, which has been attributed to the absorption of thromboplastic cell products from necrosing tumour. We conceived that the enhancement of metastasis by prior WBI may be associated with a minor degree of hypercoagulability of the blood or lymph, induced by the expression of radiation damage by lethally irradiated cells. This hypothesis has the attraction of accommodating our finding that this effect of WBI is long-lasting. In the following experiment we simulated continual absorption of thromboplastic material by repeated treatment of mice with LI tumour cells before and after excision of i.d. tumours.

Two groups of 14 mice received i.d. transplants of tumour cells and the resulting tumours were excised 24 days later. One group received i.p. injections of $\sim 10^6$ LI CBA tumour cells, or sand-ground extract of tumour, on the 5th, 11th, 14th, 21st, 24th, 26th and 32nd day after tumour transplantation. After exclusion of 6 mice which developed local recurrences, the incidences of RNM in treated and untreated mice respectively were 6/13 (mean latent period 54 days) and 2/9 (mean latent period 54 days). The difference of incidence is not significant; however, the incidence of 6/13 in the treated group is significantly greater ($P < 0{\cdot}01$) than in the pooled controls. This slight evidence of enhancement by treatment with LI cells, not reflected in a shortening of the latent period, is insufficient to support the hypothesis instigating the experiment. The absorption of thromboplastic material from the injected LI cells in this experiment is certainly greater than that to be expected from sporadic cell death in mice exposed to WBI 3 months earlier, at which time the enhancement of RNM was represented by an incidence of 12/13 and a mean latent period of 42 days (Table I).

### DISCUSSION

Our experimental design enables us to study influences on all phases of metastasis : dissemination from the primary implant, seeding of cells in remote sites and progressive growth of seeded cells. Although RNM was the least common presentation of post-operative secondary disease in our system, we have directed attention to it because it is the least studied experimentally, yet the most common in clinical cancer.

Our demonstration that pre-exposure of mice to WBI enhances RNM in this system was a chance finding. We had undertaken to study possible interaction between an allografted and an isografted tumour present simultaneously in the same animal. The mice had been previously exposed to WBI to permit growth of the allograft, but the experiment was vitiated by the unexpected high incidence of RNM from the isograft.

The current preoccupation of experimental oncologists with tumour immunity has encouraged the uncritical attachment of immunological interpretations to the results of experiments in which growth or spread of tumour has been enhanced or restrained by systemic changes in the host. Hence, attribution of our finding to the immunosuppressive effect of WBI is liable to go unquestioned. However, the hypothesis requires evidence that the tumour we have used is immunogenic. Our experience of the system has failed to reveal such evidence: putative "immunization" of mice with two inocula of LI homologous cells failed to increase the number of viable cells required to initiate tumours; 60% of mice which have been putatively "immunized" by growth and excision of i.d. tumours go on to develop progressive secondary disease; and addition of a large preponderance of LI

homologous cells to small inocula of viable cells did not reduce, but dramatically increased, the sucess of grafting (Hewitt, Blake and Porter, 1973).

Our failure to reduce the enhancing effect of WBI by early restitution of the irradiated mice with large doses of normal lymphocytes, is also discouraging to an immunological interpretation of the effect (Taliaferro, Taliaferro and Jaroslow, 1964). Our results indicated that the enhancement of RNM by WBI was quite undiminished when an interval of 3 months was allowed to elapse between irradiation and primary implantation of tumour cells, whereas immune reactivity would be expected to recover well within that period. Smith and Hollcroft (1960) reported that mice exposed to 700 rad WBI were rendered fully receptive to allografted ascites tumour cells for up to 5 days after irradiation, after which there was a gradual reassertion of resistance; by 30–44 days all the pre-irradiated mice resisted $10^6$ allografted cells.

In our attempts to demonstrate enhancement of RNM by local pre-irradiation of the lymph node, we sought some analogy between our finding and the observation that pre-irradiation of the lung increases the yield of lung nodules from i.v.-injected tumour cells (Withers and Milas, 1973; Brown, 1973). The experiments recorded in Table III showed that pre-irradiation of the nodes to a dose of 510 or 1000 rad failed to enhance RNM. The effects of lung pre-irradiation and of WBI were distinguished also by a difference in their duration: in both the above investigations it was found that the enhancement of lung-nodule formation lasted for only 30 days after irradiation, whereas the effect of WBI on RNM, as reported here, is sustained for over 90 days.

Enhancement of tumour growth by previous exposure of animals to cytolethal agents has been demonstrated by several experiments of different design to that used here. Exposure of mice to WBI one day before injection of tumour cells reduced the $TD_{50}$ for assays of viable cells of 5 different non-immunogenic tumours of spontaneous origin (Hewitt *et al.*, 1976). An important contribution to the interpretation of this finding was made by Peters (1975) using the tumour employed in the experiments reported here. He found that the $TD_{50}$ was not reduced when cells were assayed in mice which had been thymectomized and exposed to WBI 2 to 3 months previously, although persistence of their immunosuppressed state was proved by their full acceptance of small inocula of allografted tumour cells. He thus proved that reduction of $TD_{50}$ for tumour cells, in mice recently exposed to WBI, was not, in this case, an immunological phenomenon. Van Putten *et al.* (1975) demonstrated a significant decrease of RNM and increase of pulmonary metastasis, following intratesticular injection of non-immunogenic sarcoma cells, if the mice received a single dose (250 mg/kg) of cyclophosphamide 2 h after injection of tumour. In their system, pre-exposure of mice to WBI increased pulmonary metastases *but not RNM* (a notable distinction from our finding).

The experiments we have reported here have not succeeded in disclosing the mechanism of enhancement of RNM by pre-exposure of mice to WBI. We believe that further studies should take account of the most striking feature of the phenomenon: its long persistence. In this, it has some resemblance to the tumour-bed effect of irradiation.

Almost all long-persistent or late effects of irradiation, evident after acute effects have been recovered from, have been reasonably attributed to "storage" of lethal radiation damage in cells of slow turnover tissues. Our failure to demonstrate enhancement of RNM by local pre-irradiation of the tumour bed or node, indicates that the effect was not due to expression of stored damage in locally resident cells, but must involve persistent abscopal effects of radiation damage registered elsewhere. The question arises whether the enhanced nodal metastasis is due to interaction in the node between lymphatically embolized tumour cells and migrant longlived lymphocytes whose stored damage is forced to expression by the interaction.

We are grateful to Miss Angela Walder, A.I.A.T., for breeding and care of the mice. The cost of the research was met exclusively by the Cancer Research Campaign.

### REFERENCES

BENNINGHOF, D. L., TYLER, R. W. & EVERETT, N. B. (1969) Repopulation of Irradiated Lymph Nodes by Recirculating Lymphocytes. *Radiat. Res.*, **37**, 381.

BROWN, J. M. (1973) The Effect of Lung Irradiation on the Incidence of Pulmonary Metastases in Mice. *Br. J. Radiol.*, **46**, 613.

HEWITT, H. B. (1966) The Effect on Cell Survival of Inhalation of Oxygen under High Pressure during Irradiation *in vivo* of a Solid Mouse Sarcoma. *Br. J. Radiol.*, **39**, 19.

HEWITT, H. B. & BLAKE, E. R. (1968) The Growth of Transplanted Tumours in Pre-irradiated Sites. *Br. J. Cancer*, **22**, 808.

HEWITT, H. B. & BLAKE, E. R. (1971) Effect of Induced Host Anaemia on the Viability and Radiosensitivity of Murine Malignant Cells *In vivo*. *Br. J. Cancer*, **25**, 323.

HEWITT, H. B. & BLAKE, E. R. (1977) Further Studies of the Relationship between Lymphatic Dissemination and Lymphnodal Metastasis Using Non-immunogenic Murine Tumours. *Br. J. Cancer*, **35**, 415.

HEWITT, H. B., BLAKE, E. R. & PORTER, E. H. (1973) The Effect of Lethally Irradiated Cells on the Transplantability of Murine Tumours. *Br. J. Cancer*, **28**, 123.

HEWITT, H. B., BLAKE, E. R. & WALDER, A. S (1976) A Critique of the Evidence for Active Host Defence Against Cancer Based on Personal Studies of 27 Murine Tumours of Spontaneous Origin. *Br. J. Cancer*, **33**, 241.

MILAS, L. & WITHERS, H. R. (1970) Increased Incidence of Tumor Colonies in Irradiated Lungs a Transient Phenomenon. *IV Int. Cong. Radiat. Res. Evian.* Abstract 566.

PETERS, L. J. (1975) Enhancement of Syngeneic Murine Tumour Transplantability by Whole Body Irradiation—A Non-immunological Phenomenon. *Br. J. Cancer*, **31**, 293.

PETERS, L. J. & HEWITT, H. B. (1974) The Influence of Fibrin Formation on the Transplantability of Murine Tumour Cells: Implications for the Mechanism of the Révész Effect. *Br. J. Cancer*, **29**, 279.

SMITH, W. W. & HOLCROFT, J. (1960) Recovery of Radiation-induced Tolerance to Homologous Tumor. *Radiat. Res.*, **13**, 547.

TALIAFERRO, W. H., TALIAFERRO, L. G. & JAROSLOW, B. N. (1964) *Radiation and Immune Mechanisms.* New York and London: Academic Press. p. 65.

VAN PUTTEN, L. M., KRAM, L. K. J., VAN DIERENDONK, H. H. C., SMINK, T. & FÜZY, M. (1975) Enhancement by Drugs of Metastatic Lung Nodule Formation after Intravenous Tumour Cell Injection. *Int. J. Cancer*, **15**, 588.

WINTROBE, M. M. (1951) In *Clinical Hematology*, 3rd. Edn. London: Henry Kimpton. p. 325.

WITHERS, H. T. & MILAS, L. Influence of Pre-irradiation of Lung on Development of Artificial Pulmonary Metastases of Fibrosarcoma in Mice. *Cancer Res.*, **33**, 1931.